# Rapid change in host specificity in a field population of the biological control organism *Pasteuria penetrans*

**DOI:** 10.1111/eva.12750

**Published:** 2018-12-31

**Authors:** Chang Liu, Amanda Kyle Gibson, Patricia Timper, Levi T. Morran, R. Scott Tubbs

**Affiliations:** ^1^ Department of Plant Pathology University of Georgia Tifton Georgia; ^2^ Department of Biology Emory University Atlanta Georgia; ^3^ USDA ARS Tifton Georgia; ^4^ Crop and Soil Sciences, College of Agricultural and Environmental Sciences University of Georgia Tifton Georgia

**Keywords:** biological control, coevolution, crop rotation, evolution of specificity, *Meloidogyne*, *Pasteuria*, peanut, root‐knot nematode

## Abstract

In biological control, populations of both the biological control agent and the pest have the potential to evolve and even to coevolve. This feature marks the most powerful and unpredictable aspect of biological control strategies. In particular, evolutionary change in host specificity of the biological control agent could increase or decrease its efficacy. Here, we tested for change in host specificity in a field population of the biological control organism *Pasteuria penetrans*. *Pasteuria penetrans* is an obligate parasite of the plant parasitic nematodes *Meloidogyne *spp., which are major agricultural pests. From 2013 through 2016, we collected yearly samples of *P. penetrans* from eight plots in a field infested with *M. arenaria*. Plots were planted either with peanut (*Arachis hypogaea*) or with a rotation of peanut and soybean (*Glycine max*). To detect temporal change in host specificity, we tested *P. penetrans *samples annually for their ability to attach to (and thereby infect) four clonal lines of *M. arenaria*. After controlling for temporal variation in parasite abundance, we found that *P. penetrans *from each of the eight plots showed temporal variation in their attachment specificity to the clonal host lines. The trajectories of change in host specificity were largely unique to each plot. This result suggests that local forces, at the level of individual plots, drive change in specificity. We hypothesize that coevolution with local *M. arenaria *hosts may be one such force. Lastly, we observed an overall reduction in attachment rate with samples from rotation plots relative to samples from peanut plots. This result may reflect lower abundance of *P. penetrans* under crop rotation, potentially due to suppressed density of host nematodes. As a whole, the results show local change in specificity on a yearly basis, consistent with evolution of a biological control organism in its ability to infect and suppress its target pest.

## 1 INTRODUCTION

Host specificity is the foremost challenge for the safe and effective use of biological control agents (reviewed in Brodeur, 2012; Fagan, Lewis, Neubert, & Driessche, 2002; McEvoy, 1996). Upon release into a novel range, generalist predators or parasites may attack unintended prey or host species, leading to a decline in their population size and an increase in extinction risk (e.g., Boettner, Elkinton, & Boettner, 2000; Louda, Kendall, Connor, & Simberloff, 1997; reviewed in Louda, Pemberton, Johnson, & Follett, 2003; Simberloff & Stiling, 1996). Such nontarget effects motivated a shift toward biological control agents with narrow host ranges. Efforts to identify these more specialized candidates include extensive prescreening of potential host ranges and a focus on native enemies, for which the host range within the local community is known and likely limited by a prior evolutionary history (Brodeur, 2012; Greathead, 1995; Roderick, Hufbauer, & Navajas, 2012; Secord & Kareiva, 1996; Waage, 2001). Narrow host ranges bring their own set of limitations, however. If a population of a biological control agent is specialized to attack only a subset of genotypes within a single host species, we would predict limited efficacy against the majority of host populations (Parker, 1985) (reviewed in Brodeur, 2012).

In theory, rapid evolution can ameliorate the problem of high specificity in biological control. Indeed, a major proposed advantage of biotic over abiotic control is that biological antagonists can rapidly adapt as host populations evolve (e.g., Dwyer, Levin, & Buttel, 1990; Hajek, Humber, & Elkinton, 1995; reviewed in Hopper, Roush, & Powell, 1993; Roderick & Navajas, 2003). The field of host–parasite coevolution provides a valuable theoretical framework for predicting evolutionary trajectories. Coevolutionary theory predicts that parasites adapt to infect locally common host genotypes (Haldane, 1949), and several studies support this prediction (Chaboudez & Burdon, 1995; Koskella & Lively, 2009; Lively & Dybdahl, 2000; Wolinska & Spaak, 2009). Thus, high host specificity may not prevent a parasite from serving as an effective biological control. With sufficient selection and genetic variation, a population of parasites applied as biological control could adapt to infect and suppress a diversity of host genotypes across populations (Gandon, 2002; Gandon & Michalakis, 2002). Clearly, the potential for rapid evolution of host specificity is a significant factor to consider in evaluation of biological control agents. Yet there exists little evidence of the evolution of specificity in biological control systems (though see Le Masurier & Waage, 1993; Salt & van den Bosch, 1967) (reviewed in Hufbauer & Roderick, 2005; van Klinken & Edwards, 2002).

Here, we tested for temporal change in host specificity in the bacterial parasite *Pasteuria penetrans*. *Pasteuria penetrans* is an obligate parasite of root‐knot nematodes (*Meloidogyne *spp.) (Mankau, 1975; Sayre & Starr, 1985; Sayre & Wergin, 1977). *Meloidogyne* spp. infect numerous crop plants, particularly in tropical and subtropical regions of the world (Sasser, 1977). They establish permanent feeding sites in plant roots, siphoning nutrients from the plant and impairing root function (reviewed in Moens, Perry, & Starr, 2009; Sasser & Carter, 1985; Sasser, Eisenback, & Carter, 1983; Trudgill & Blok, 2001). This results in billions of dollars in lost yield each year (Nicol et al., 2011; Onkendi, Kariukib, Maraisc, & Molelekia, 2014). Accordingly, Jones et al. (2013) named *Meloidogyne *spp. the most economically important plant parasitic nematodes today. The focus of this study is *Meloidogyne arenaria*, the peanut root nematode, a polyploid asexual species responsible for significant crop damage in the southeastern United States (Blanc‐Mathieu et al., 2017; Ingram & Rodriguez‐Kabana, 1980; Motsinger, Crawford, & Thompson, 1976; Starr & Morgan, 2002; Wheeler & Starr, 1987).

*Pasteuria penetrans* offers a biological alternative to chemical controls, which are expensive, toxic, and increasingly difficult to obtain (Onkendi et al., 2014). Endospores of *P. penetrans *attach to the cuticles of juvenile nematodes as they migrate through the soil in search of roots. Once a nematode establishes within a root, the attached bacterium penetrates the cuticle and develops vegetatively within the host body (Sayre & Wergin, 1977). Females infected with *P. penetrans* produce few to no offspring (Bird, 1986; Bird & Brisbane, 1988). Application of *P. penetrans *to plots can dramatically reduce nematode densities and increase crop yield (Brown, Kepner, & Smart, 1985; Mankau, 1975, 1980; Mankau & Prasad, 1972). As a result, *P. penetrans *is under consideration as a commercial product for control of *Meloidogyne* spp. A related species, *P. nishizawae*, is already being marketed to control the soybean cyst nematode *Heterodera glycines*.

Prior studies suggest that genotypes of *P. penetrans *are highly specialized, infecting a narrow subset of host genotypes. A single population of *P. penetrans *varies dramatically in its potential to infect different host populations (Davies, Kerry, & Flynn, 1988; Duponnois, Fargette, Fould, Thioulouse, & Davies, 2000; Spaull, 1984; Stirling, 1985). For example, when populations of *M. incognita* in Ecuador were challenged with a single population of *P. penetrans*, the frequency at which endospores attached to the cuticles of hosts from different populations ranged from <10% to >90% (Trudgill et al., 2000). In addition, a single population of *P. penetrans* contains a diversity of genotypes that vary in their host specificity (Davies, Redden, & Pearson, 1994; Joseph, Schmidt, Danquah, Timper, & Mekete, 2017; Timper, 2009). Extensive study of *P. ramosa, *a related parasite of the cladoceran *Daphnia magna*, indicates that high host specificity may be a feature of this genus. Infection success of *P. ramosa* varies with its ability to attach to the cuticle in the host esophagus (Duneau & Ebert, 2012; Duneau, Luijckx, Ben‐Ami, Laforsch, & Ebert, 2011; Ebert et al., 2016). Switching the identity of a single host allele can render a resistant host genotype susceptible, likely by enabling parasite attachment (Luijckx, Fienberg, Duneau, & Ebert, 2012, 2013; Metzger, Luijckx, Bento, Mariadassou, & Ebert, 2016). Similarly, the interaction of proteins on the spore surface and the nematode cuticle may mediate attachment and, thereby, specificity of *P. penetrans* (Davies, 1994, 2009; Davies et al., 1994). There is some evidence that specificity can evolve in this system (Oostendorp, Dickson, & Mitchell, 1990; Timper, 2009; Tzortzakakis & Gowen, 1994). Channer and Gowen (1992) reared a population of *P. penetrans *on three novel host populations. In one case, they found that the parasite population increased in its ability to attach to the novel host population with which it was reared. It simultaneously lost its ability to attach to its original host population. Hosts can also evolve resistance: glasshouse experiments showed a decrease in attachment rate as a host population was continually challenged with a static parasite population (Tzortzakakis, Gowen, & Goumas, 1996), with the evolved resistance specific to the parasite population in the experiment (Tzortzakakis & Gowen, 1994). A fundamental question remains as follows: Does host specificity rapidly change in field populations of this parasite?

We addressed this question by testing for spatial and temporal variation in the host specificity of *P. penetrans *sampled from plots in an experimental agricultural field. Long‐term sampling of this study site provided preliminary evidence for change in host specificity: Starting in 1998, we used a single laboratory population of *M. arenaria *to assay the soil for abundance of *P. penetrans *endospores. Large numbers of endospores attached to assayed nematodes in 1998 and 1999. In subsequent years, the numbers of attached endospores declined. Repeating the assay with different host lines revealed that this decline in attachment did not occur because endospores had disappeared from the soil, but because the *P. penetrans *population had lost the ability to attach to the standard laboratory population of *M. arenaria *(Timper, 2009). We hypothesized that *P. penetrans *populations evolve rapidly in their host specificity. To test this hypothesis, we sampled endospores from eight plots of the experimental field from 2013 to 2016. For each plot, we measured variation in host specificity by quantifying the attachment rate of each endospore sample to four clonal lines of *M. arenaria*. After controlling for variation in endospore abundance, we observed substantial yearly change in attachment rates of field‐sampled *P. penetrans* to the tested host lines, consistent with the hypothesis of rapid evolution of host specificity.

## 2 MATERIALS AND METHODS

### 2.1 Natural history

The genus *Meloidogyne *is diverse and globally distributed. Many species are diploid, exhibit a range of reproductive strategies, and infect a narrow range of noncrop plant species. However, the most widespread and destructive species reproduce asexually (Castagnone‐Sereno, Danchin, Perfus‐Barbeoch, & Abad, 2013; Chitwood & Perry, 2009; Triantaphyllou, 1985, 1991). Three prominent crop pests, *M. incognita, M. javanica, *and *M. arenaria*, reproduce exclusively via mitotic parthenogenesis and have elevated ploidy (triploid, tetraploid, and tetra‐ to pentaploid, respectively). This complex of closely related parthenogens likely arose from multiple hybridization events (Blanc‐Mathieu et al., 2017). Here, we focus on *M. arenaria*, which parasitizes a diversity of plant hosts, including peanuts, cucurbits, soybean, potato, tobacco, tomato, peach, and eggplant (reviewed in CABI, 2017; Onkendi et al., 2014).

The life cycle of *Meloidogyne* spp. takes three to six weeks to complete and begins as eggs in the soil. Second‐stage juveniles (J2) hatch from the eggs and migrate through soil in search of host plants. A J2 infects root tips and establishes a permanent feeding site, where it siphons nutrients from nearby plant cells via a feeding tube. The J2 passes through two additional juvenile stages before molting into a mature female. As the nematode develops, a gall forms around it due to enhanced growth and replication of the surrounding plant cells. The female deposits eggs into a gelatinous matrix, which can facilitate the movement of the eggs to the exterior surface of the gall (Moens et al., 2009).

*Pasteuria *sp. are gram‐positive, endospore‐forming bacteria (Mankau, 1975; Sayre & Starr, 1985; Sayre & Wergin, 1977; Starr & Sayre, 1988). Members of the genus naturally parasitize a diversity of nematodes (Chen & Dickson, 1998; Sayre & Starr, 1985), excepting *P. ramosa, *which parasitizes cladocerans (Ebert, Rainey, Embley, & Scholz, 1996; Metchnikoff, 1888). *Pasteuria *endospores resist environmental stress (e.g., desiccation) (Williams, Stirling, Hayward, & Perry, 1989) and can retain viability for multiple years in the laboratory (Espanol, Verdejo‐Lucas, Davies, & Kerry, 1997; Giannakou, Pembroke, Gowen, & Davies, 1997; Mani, 1988). Endospores of *P. penetrans *first enter the soil upon decomposition of the parasitized nematode and plant root. J2s acquire endospores as they migrate. Though a single spore suffices for an infection to establish, a J2 can acquire many endospores. Attachment of multiple endospores can impair J2 mobility, preventing it from finding and establishing in a plant root (Stirling, 1984). Germination of attached endospores depends upon cues associated with establishment of the J2 host in the root. The endospore produces a germ tube that penetrates the host cuticle, entering the pseudocoelom. The bacterium then develops and proliferates, reducing or eliminating host reproduction in the process (Mankau & Imbriani, 1975; Phani & Rao, 2018).

This study focuses on host–parasite specificity at the attachment stage. Variation in infection could arise at multiple stages in the life cycle of *Meloidogyne* and *P. penetrans *(for full life cycle diagram, see Figure 1 of Preston et al., 2003). Successful infection requires that a *Pasteuria *bacterium persist in soil, make contact with a J2 host, attach to the host's cuticle, penetrate the cuticle to enter the host's body cavity, overcome within‐host defenses (Tarr, 2012), and reproduce. Each of these steps may be influenced by host genetics, parasite genetics, environmental factors, and their interactions (Ebert et al., 2016; Kruitwagen, Beukeboom, & Wertheim, 2018). For example, endospore attachment varies with temperature, pH (Chen & Dickson, 1998), and plant root exudates (Liu, Timper, Ji, Mekete, & Joseph, 2017). In addition, replication rate of *P. penetrans* increases with temperature (Chen & Dickson, 1998; Lopes, Orr, & Blok, 2018). In spite of all this variation, it is possible to identify steps at which host and parasite are most likely to respond to selection: Experiments spanning the infection process for *P. ramosa *on *Daphnia *hosts highlight attachment as the infection step most strongly influenced by the genetic interaction of host and parasite. Variation in the attachment step is thus most likely to drive coevolutionary interactions for *P. ramosa* (Duneau et al., 2011; Ebert et al., 2016; Luijckx, Ben‐Ami, Mouton, Pasquier, & Ebert, 2011). It remains to be determined whether these findings also apply to *P. penetrans*–nematode interactions.

**Figure 1:**
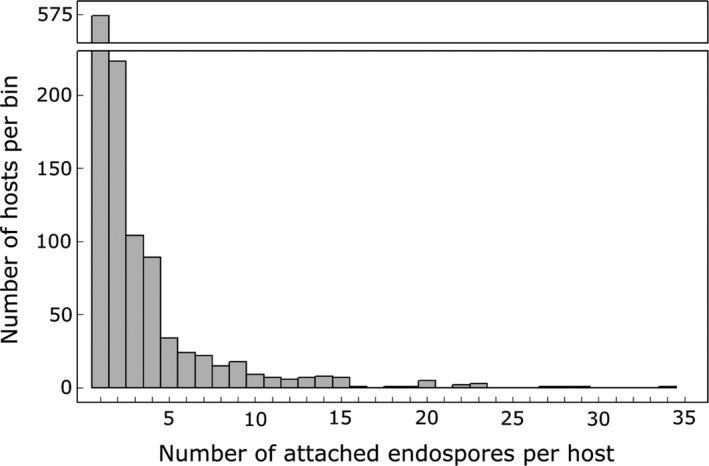
Distribution of endospore counts per host. The histogram is restricted to the 1,164 J2 nematode hosts that had one or more endospores of *Pasteuria penetrans *attached. Data shown for 1,164 J2 nematode hosts

### 2.2 Experimental site and design

The study site was a 0.77 hectare agricultural field at the University of Georgia Gibbs Farm, Tifton, Georgia, USA, that was naturally infested with *M. arenaria* race 1 and *P. penetrans*. The soil was a Tifton loamy sand (fine‐loamy, siliceous, thermic Plinthic Kandiudult; pH 6.1). The site contained a crop rotation experiment where the primary crop was peanut. There were 17 rotation sequences (treatments) arranged in a randomized complete block design with four replications. Samples for *P. penetrans* were collected from replicate plots of two treatments in the experiment: peanut (cv. GA‐06G) rotated with soybean (cv. Pioneer 95Y20) (P‐S‐P) and continuous peanut (P‐P‐P). Both peanut and soybean are good hosts for *M. arenaria.* The plot dimensions and locations of sampled plots are shown in Supporting information Figure S1.

In the spring, the soil was plowed to a depth of 20 to 25 cm before shaping into planting beds 1.8 m wide and 10 to 15 cm high. Each plot included four beds. The field was planted with a new seed lot each year. Both peanut and soybean were planted in May with seeds spaced in two rows, 0.9 m apart on the bed, with 20 seed/m for peanut and 24 seed/m for soybean. For the P‐S‐P rotation, soybean was planted in 2012, 2014, and 2016. Crop management, including fertilization and pesticides, were conducted based on recommendations for the area (Guillebeau, 2009). Crops were harvested at optimum maturity in mid‐September to late September.

### 2.3 Field sampling and bioassay of specificity

Four clonal lines of *M. arenaria *(C3, C6, C8, and C40) previously found to differ in patterns of endospore attachment were used to assay *P. penetrans* endospores from subsamples of soil from each plot. The clonal lines were obtained in 2008 by picking single egg masses from peanut growing in field soil infested with the nematode (Timper, 2009). They were maintained on eggplant (*Solanum melongena*, cv. Black Beauty) in a glasshouse at 22–30°C. We also assayed *P. penetrans *endospores using hosts from a glasshouse line (GH) of *M. arenaria *that had been originally isolated from Gibbs Farm in the early 1990 s. This line was maintained on tomato (*Solanum lycopersicum *L. cv. Rutgers) and eggplant in the glasshouse. In this study, we focus on data collected from the four clonal host lines, except in the case of one analysis. Hosts for the attachment assay were obtained by placing roots with egg masses separately in a mist chamber. The hatched J2 were collected 3–4 days later.

Soil for assaying *P. penetrans* specificity was collected from the center of two beds of each plot in early October from 2013 to 2016. For each plot, 10 root‐zone soil cores (2.5 cm diam; 15 cm deep) were collected in each row and mixed thoroughly to obtain a large, representative sample of endospores from the plot. All soil was heated at 60°C for 2 hr before use to kill the native *M. arenaria*. Specificity was measured using a bioassay previously described by Timper et al. (2001). In this assay, the four clonal host lines served as probes to detect shifts in specificity of *P. penetrans *in the field. A subsample of 100 cm^3^ soil was added along with tap water to a flask, and the flask was shaken vigorously to make a slurry before decanting the soil–water suspension into another 250‐ml flask. Second‐stage juveniles (1,500 J2) of one clonal line of *M. arenaria* were added to the soil–water suspension and shaken on a rotary shaker at 150 rpm. After 24 hr, the J2s were extracted by centrifugal floatation (Jenkins, 1964), and the number of endospores adhering to 25 randomly selected J2s was determined at 400× magnification with an inverted microscope. For each soil sample, we repeated this bioassay for all four clonal host lines. The relative differences in attachment rate to J2s of the four clonal host lines provided a quantitative estimate of the host specificity of the tested parasite sample. Similar approaches are used for evaluating specificity in host–parasite interactions, including for *Ustilago bullata* and cheatgrass (*Bromus tectorum*) (Meyer et al., 2005), *Colletotrichum lindemuthianum* and common bean (*Phaseolus vulgaris*) (Sicard, Michalakis, Dron, & Neema, 1997), *Melampsora lini* and Australian flax (*Linum marginale*) (Thrall et al., 2012), and *Pasteuria ramosa *and water fleas (*Daphnia magna*) (Luijckx et al., 2011).

### 2.4 Statistical analyses

For the following analyses, we converted spore counts per host to a binomial variable: 0 for zero endospores attached and 1 for one or more endospores attached. This conversion enabled us to broadly compare hosts that could potentially be parasitized (endospores attached) to those that could not (no endospores attached). All models were fit to this binomial response variable using a logit link function. All analyses were performed in R v3.3.1 (R Core Team, 2013).

Both the abundance and the specificity of *P. penetrans *endospores in tested soil may contribute to variation in the rates of endospore attachment to tested hosts. For example, attachment rates could be elevated in a particular assay if the parasite population size is large (i.e., abundance or dose) or if alleles conferring ability to attach to the tested host line are at high frequency in the parasite population. We used a statistical approach that enabled us to test for changes in specificity by controlling for the contribution of endospore abundance to variation in attachment rates. Specifically, we included main effects (e.g., year, plot) and, where relevant, a two‐way interaction (i.e., plot × year) to control for intrinsic variation between parasite sources. Abundance of parasite endospores is an intrinsic difference between parasite sources; for example, attachment rates will be relatively high from soil drawn from a plot with high parasite abundance, regardless of the host line used. In this case, including plot as a main effect in the model controls for this difference in endospore abundance. After controlling for differences in endospore abundance with these statistical terms, we could use the remaining interactions with host line to test for changes in specificity, which we describe further in the subsection below entitled “*Change in host specificity.”*

#### 2.4.1 Variation in endospore abundance

We first evaluated variation in endospore abundance. Specifically, we compared endospore abundance in soils collected from the two crop treatments. We fit a generalized estimating equation (GEE) with year (2013–2016), clonal line (C3, C6, C8, C40), treatment (peanut, rotation), and all possible interactions as predictors of the probability of endospores attaching to a host. The response variable was the number of hosts with and without endospores attached in a tested batch of 25 hosts. We used the GEE framework to cluster batches of hosts according to the plot from which the tested soil was sampled. A first‐order autoregressive correlation structure between batches from the same plot was selected due to the longitudinal nature of the sampling (Wang & Carey, 2003; Ziegler & Vens, 2010). The results of Wald tests dictated the exclusion of insignificant interaction effects (Zuur, Ieno, Walker, Saveliev, & Smith, 2009).

The host clonal lines used in the bioassays were collected in 2008 from the peanut plots that we surveyed from 2013 to 2016. This shared origin raised the possibility that endospores sampled from peanut plots had greater ability to attach to the host clonal lines than did endospores sampled from rotation plots. It is unlikely that parasite samples from interspersed peanut and rotation plots would strongly differ in adaptation to this small sample of host genotypes. Nonetheless, with the above analysis, we cannot conclusively attribute differences in attachment rates between treatments to differences in endospore abundance between treatment plots. To address this problem, we repeated the analysis using hosts from an independent lineage (GH), which was established in the early 1990s. This host line does not share a coevolutionary history with parasites from either the peanut or rotation plots. Hence, we can attribute variation in attachment rate between treatments to variation in endospore abundance between treatments. We fit a GEE as described above with year (2013–2016), treatment (peanut, rotation), and their interaction as predictors of the probability of endospores attaching to a host.

#### 2.4.2 Change in host specificity

We then evaluated changes in the specificity of *P. penetrans* through time. Peanut and rotation plots were analyzed in two separate models. For each treatment, we fit a logistic model (generalized linear model—GLM) with year, clonal line (C3, C6, C8, C40), plot, and all possible interactions as predictors of the probability of endospores attaching to a host. The response variable was the attachment status (0, 1) of an individual host. For these two models, the predictors’ year, plot, and their interaction controlled for variation in endospore abundance. After controlling for differences in endospore abundance, we tested for differences in specificity using two‐way and three‐way interactions of year, line, and plot. The interaction “line × plot” addressed spatial variation in attachment rate to host lines, across all years. A substantial line × plot interaction would indicate static differences between plots in specificity. The interaction “year × line” addressed temporal variation in attachment rate to host lines, shared across plots. A substantial year × line interaction would indicate change in specificity, with the temporal trajectory similar across plots. The interaction “year × line × plot” simultaneously addressed spatial and temporal variation in specificity. A significant three‐way interaction would indicate change in specificity, with the temporal trajectories differing between plots.

In an effort to weigh the relative importance of model terms, we compared deviance values from the model outputs. Deviance values are not a measure of variance explained, as in ordinary least squares regression, but they provide a sense of how much of the goodness of fit of the overall model is attributable to individual predictors. For these comparisons, we used the average of deviance values obtained by entering a given term at all possible positions in a model. We estimated McFadden's pseudo‐*R^2^* to quantify the explanatory power of the final model. Values of this estimate can range widely based upon the nature of the dataset, but values between 0.2 and 0.4 are considered indicative of strong explanatory power (McFadden, 1974, 1979).

Lastly, we repeated the above analysis at the level of each individual plot, for a total of eight logistic models. For each model, the predictors were year, clonal line (C3, C6, C8, C40), and their interaction. The models were otherwise as described for the prior analysis. In these eight models, the predictor year controlled for yearly variation in the abundance of endospores in soil sampled from the focal plot. Controlling for differences in endospore abundance, we could then test for temporal change in specificity using the interaction term. A substantial interaction effect would indicate change in specificity.

## 3 RESULTS

### 3.1 Endospore attachment

From 2013 to 2016, we tested a total of 4,000 *M. arenaria *J2 hosts from four clonal lines for attachment by endospores of the natural parasite *P. penetrans* in soils from eight plots*. *For each tested host, we counted the number of attached endospores. Across all trials, *P. penetrans *endospores attached to a mean of 36.4% ± 2.2% (standard error of the mean, SEM) of the hosts. Of those hosts with endospores attached, the median number of attached endospores was two. The majority of hosts had just one (49.3%) or two (19.2%) endospores attached (Figure 1). Only 12% had more than five and 1.5% (17 hosts) more than 15. Thus, the number of attached endospores and the variation in number between hosts were relatively low. For the remainder of the analyses, we investigated the binary outcome of endospore attachment (yes/no). This approach enabled us to draw a clear distinction between hosts that could potentially be parasitized (endospores attached) and those that could not (no endospores attached).

### 3.2 Variation in endospore abundance

Four of the eight study plots were continuously planted with peanut from 2013 to 2016. For the other four study plots, the crop alternated annually between peanut and soybean. We refer to these as the peanut and rotation treatments, respectively.

**Figure 2:**
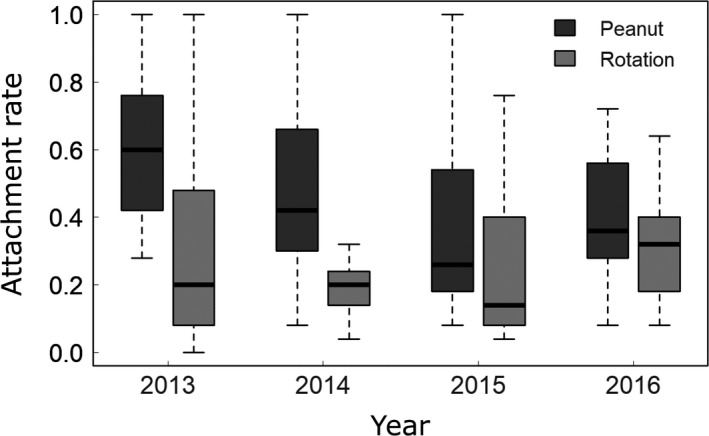
Endospore attachment rates by treatment. Endospore attachment rates were higher with soils collected from peanut plots vs. rotation plots. Attachment rate was calculated as the fraction of 25 tested hosts with endospores attached. Each boxplot represents 16 estimates of attachment rate (four clonal lines by four plots/treatment)

We first compared rates of endospore attachment between soils derived from plots subjected to these two crop treatments. A significantly higher fraction of tested hosts acquired endospores when exposed to soils from peanut plots (46.1% ± 3.0%) relative to rotation plots (26.6% ± 2.7%) (Figure 2, Table 1a). Repeating this analysis using a distinct host line (GH) gave the same result: A significantly higher fraction of GH hosts acquired endospores when exposed to soils from peanut plots (42.2% ± 4.2%) relative to rotation plots (21.3% ± 4.1%) (Table 1b). These results suggest the maintenance of a higher abundance (i.e. dose) of endospores in soils of peanut plots: Attachment rates were higher with soils from peanut plots, regardless of the host lines tested.

**Table 1:** Variation in endospore attachment rate across crop rotation treatments

(a) Four clonal host lines				(b) Glasshouse line			
	*df*	χ^2^	*p*‐value		*df*	χ^2^	*p*‐value
Year	3	5.51	0.138	Year	3	1.65	0.637
Line	3	3.40	0.334	Treatment	1	12.31	<0.001
Treatment	1	25.41	<0.001	Terms excluded based upon Wald test			
Year × Line	9	25.82	0.002	Year × Treatment	3	1.30	0.730
Year × Treatment	3	11.04	0.012				
Terms excluded based upon Wald test							
Line × Treatment	3	5.70	0.130				
Year × Line × Treatment	9	6.40	0.700				

Notes

These tables present the results of generalized estimating equations with the number of hosts with and without attached endospores as a binomial response variable. The Wald chi‐square statistics were obtained by sequentially adding each factor and comparing models with and without the factor of interest.

**Figure 3:**
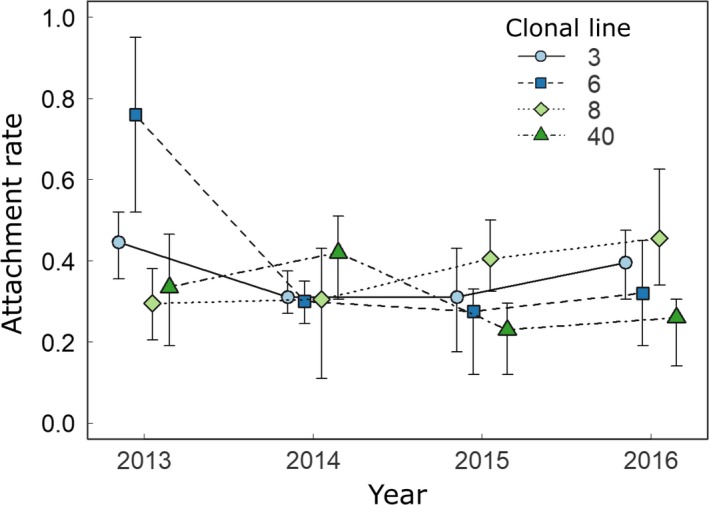
Mean attachment rates through time. For each line, the graph shows the mean attachment rate across the eight plots surveyed each year. Attachment rates were estimated using 25 hosts for each line‐by‐plot‐by‐treatment combination. Error bars are 95% confidence intervals for the mean calculated using the function groupwiseMean in the R package rcompanion (Mangiafico, 2015)

Mean attachment rates did not vary with year, indicating no overall variation in endospore abundance over time (Table 1, Figure 3). Attachment rates also did not vary with host clonal line, indicating that, averaging over all samples collected from the field plots, *P. penetrans *did not attach better to one host line than another (Table 1a, Figure 3). There was, however, a significant interaction of year and line, indicating that sampled *P. penetrans* varied among years in attachment rate to specific host lines (Table 1a). This effect appeared to be driven by an overall decline in the attachment rate to C6 from 76.0% ± 8.0% in 2013 to 30.0% ± 4.8% in 2014 (GEE coefficient estimate = −1.60 ± 0.67, *p* = 0.016), 27.5% ± 5.5% in 2015 (−1.72 ± 0.77, *p* = 0.025), and 32.0% ± 6.2% in 2016 (−1.88 ± 0.72, *p* = 0.009). Yearly mean attachment rates of the other lines remained relatively constant through time, at 36.5% ± 3.3% for C3, 36.5% ± 3.9% for C8, and 31.1% ± 4.2% for C40 (Figure 3). Accordingly, this interaction became insignificant if we excluded line C6 from the analysis (*df* = 6, χ^2^ = 10.93, *p* = 0.091) but remained significant if any other line was excluded.

### 3.3 Change in host specificity

We tested the hypothesis that the specificity of *P. penetrans* changed in these plots. To do so, we evaluated variation in the attachment rate of *P. penetrans* against the four clonal *M. arenaria* lines. We used a statistical approach that enabled us to test for variation in host specificity by controlling for differences in the abundance of endospores in sampled plots (see Materials and Methods). Because of the substantial differences between treatments identified above, we analyzed peanut and rotation plots separately.

For both treatments, attachment rates varied with year, plot, and their interaction (Table 2). These effects likely reflect the expected spatial and temporal variation in the abundance of *P. penetrans *endospores in tested soils. Inclusion of these predictors in the models controlled for variation in endospore abundance, allowing us to then evaluate variation in host specificity of sampled parasites.

**Table 2:** Variation in endospore attachment rate across space, time, and host line

(a) Peanut				(b) Rotation			
	*df*	*D*	*p‐value*		*df*	*D*	*p‐value*
Year	3	56.47	<0.001	Year	3	21.84	<0.001
Line	3	7.30	0.063	Line	3	37.47	<0.001
Plot	3	38.70	<0.001	Plot	3	14.26	0.003
Year × Line	9	60.99	<0.001	Year × Line	9	103.23	<0.001
Year × Plot	9	99.71	<0.001	Year × Plot	9	83.47	<0.001
Line × Plot	9	46.71	<0.001	Line × Plot	9	28.69	<0.001
Year × Line × Plot	27	123.10	<0.001	Year × Line × Plot	27	81.79	<0.001
Null deviance	1,599	2208.5		Null deviance	1,599	1854.4	
Residual deviance	1,536	1775.5		Residual deviance	1,536	1,483.6	
*R_2_^L^* = 0.196				*R_2_^L^* = 0.200			

Notes

These tables present the results of generalized linear models with the attachment status of an individual host (endospores attached or not) as a binomial response variable. The same model was separately fit to data from peanut plots and rotation plots. For each factor, we show the results of likelihood ratio tests of models with and without the factor. *D *is the deviance accounted for by each factor. *R^2^_L_*reflects the explanatory power of the model.

**Figure 4:**
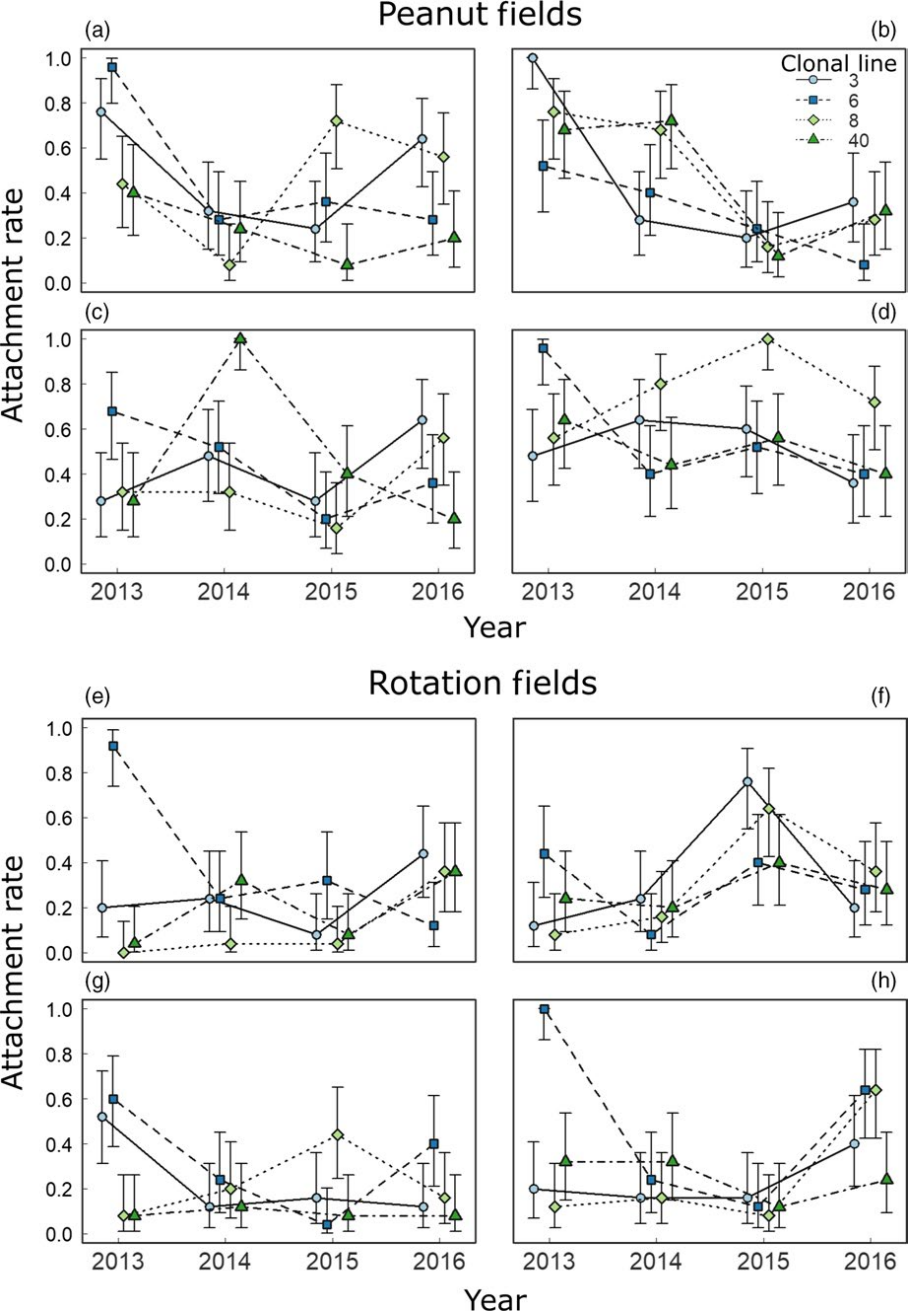
Temporal change in *Pasteuria penetrans* specificity at the local level. The fraction of hosts with attached endospores is shown for each of the four peanut (a–d) and rotation (e–h) plots. The relative attachment rates to host lines, and even their rank order, varied between years in the same plot and between plots in the same year, consistent with spatial and temporal variation in host specificity. Each data point is estimated from 25 hosts. Error bars are 95% confidence intervals for the proportion calculated using the function binom.test in R

After controlling for variation in endospore abundance, we found evidence for change in host specificity in plots of both treatments (Table 2). For peanut plots, a three‐way interaction of year, clonal line, and plot contributed strongly to variation in attachment rates (Table 2a). This interaction indicated that parasites varied through time in their ability to attach to the four host clonal lines and that these temporal trajectories varied significantly between the four peanut plots. Figure 4a–d shows four distinct trajectories of yearly change in the rank order of attachment of clonal lines, consistent with rapid change in the specificity of the parasites from each plot. The significant two‐way interaction of year and clonal line indicated that some of the temporal variation in specificity was shared across plots. The significant two‐way interaction of plot and clonal line further indicated that some of the spatial variation in specificity was fixed in time. However, comparison of deviance values (D) showed that the three‐way interaction accounted for approximately twice as much of the model's explained deviance as the two‐way interaction of year and line and more than twice as much as the two‐way interaction of plot and line. This result suggests that, in the four peanut plots, temporal change in local, plot‐level factors was the dominant driver of variation in specificity between parasites sampled from peanut plots (Table 2a).

For rotation fields, the three‐way interaction of year, clonal line, and plot also contributed to variation in attachment rates (Table 2b, Figure 4e–h). In these fields, however, the two‐way interaction of year and line contributed slightly more (1.3‐fold) to variation in attachment rates than did the three‐way interaction. This result suggests that the trajectories of host specificity were relatively similar across the four rotation plots. This finding suggests that temporal change in global, across‐plot factors were important in driving variation between parasites sampled from rotation plots.

To complement the above analyses, we investigated change in host specificity for the parasites from each individual plot. For each of the eight plots, attachment rate varied substantially with the interaction of year and clonal line after controlling for yearly differences in endospore abundance (Supporting information Table S1). This result further supports the above findings of change in specificity through time (Figure 4).

## 4 DISCUSSION

In this study, we tested for the evolution of host specificity of a parasite proposed as a biological control agent. After controlling for variation in parasite abundance, we detected yearly changes in the ability of *Pasteuria penetrans* to attach to a collection of clonal lines of its host, the nematode *Meloidogyne arenaria *(Figure 4, Table 2, Supporting information Table S1). This result is consistent with the original hypothesis: It demonstrates rapid change in specificity for host attachment, a phenotype that is fundamental to the ability of this parasite to infect its host. Temporal trajectories of host specificity differed substantially across individual plots sampled within a single field (Table 2, Figure 4). This result suggests that change in host specificity occurs at a local scale (Figure 3 vs. 4). Below, we present hypotheses for the drivers of change in host specificity in *P. penetrans* and discuss the implications of changing specificity for the use of *P. penetrans* in biological control.

Before presenting hypotheses for the drivers of changing specificity, we would like to note a few limitations of this study. First, the assay of host specificity only used four clonal host lines. Assuming a genetic basis to attachment, the assay could only detect variation at those loci and alleles associated with attachment to the four tested host lines. The assay, therefore, was conservative, and variation in host specificity likely exceeds that shown here. Second, the assay measured variation in attachment of *P. penetrans* to *M. arenaria*. Attachment does not ensure a successful infection (Davies et al., 1988; Oostendorp, Dickson, & Mitchell, 1991; Sayre & Wergin, 1977; Stirling, 1984). Infection cannot, however, proceed without attachment, so variation in specificity rests upon this first step in the infection process. Moreover, Duneau et al. (2011) primarily attributed variation in specificity of the parasite *P. ramosa* to variation in attachment to *Daphnia *hosts. Third, we cannot exclude the possibility that the observed changes in host specificity are not genetic and hence not evolutionary. We measured attachment rates of endospores directly following field collection. This approach preserved the natural parasite diversity, but it did not allow for a period of propagation in the laboratory to remove any environmental effects on host specificity. Studies of *P. ramosa* demonstrate that variation in attachment rests largely upon the genetic interaction of host and parasite (Carius, Little, & Ebert, 2001; Duneau et al., 2011; Ebert et al., 2016; Luijckx, Fienberg, Duneau, & Ebert, 2013). The extent to which this is also true for *P. penetrans* remains to be determined, though prior studies are suggestive of a genetic basis (Davies et al., 1988, 1994; Espanol et al., 1997; Stirling, 1985). Hence, we find evidence of change in specificity that is consistent with, but not equivalent to, the evolution of specificity.

### 4.1 Hypothetical drivers of specificity change

We now propose and discuss three hypotheses to explain the rapid change in host specificity of *P. penetrans. *First, temporal and spatial variation in specificity may simply reflect genetic drift. Given the relatively limited dispersal distances of both *P. penetrans *(Oostendorp et al., 1990) and *Meloidogyne *spp. (Prot & Netscher, 1979)*, *patterns of random variation in allele frequencies could differ across, and even within, plots. Current data cannot reject a contribution of drift. There is, however, good reason to suspect that *P. penetrans* populations experience strong selection on their ability to attach to local hosts: Development of *P. penetrans* cannot begin without attachment (Sayre & Wergin, 1977).

Second, abiotic factors may drive the evolution of host specificity in *P. penetrans. *We predict that some abiotic variables would have similar trajectories of temporal change across the whole field (e.g., seasonal variation in temperature and precipitation). These abiotic factors should drive parallel trajectories of specificity change across plots within the field. We, however, observed substantial variation in evolutionary trajectories among plots (Figure 4). To explain this localized variation, changes in specificity must be driven by abiotic factors that vary locally, between plots. Evaluation of this hypothesis requires knowledge of the abiotic variables that might impact specificity of *P. penetrans*.

Third, the change in host specificity of *P. penetrans* may reflect negative frequency‐dependent selection. According to this hypothesis, parasite populations adapt to infect the most common genotypes in the local host population. Burdened by infection, these common host genotypes decline in frequency. As new host genotypes increase in frequency, selection on the parasite population shifts (Bell, 1982; Hutson & Law, 1981; Jaenike, 1978). Such coevolutionary cycles can arise when a genotype‐by‐genotype interaction determines infection, and host and parasite populations can rapidly adapt to one another (Engelstadter & Bonhoeffer, 2009; Hamilton, 1980; Hamilton, Axelrod, & Tanese, 1990; Parker, 1994). Prior studies suggest that the interaction of *P. penetrans* and *Meloidogyne* spp. meets these requirements. G × G interactions contribute to attachment (Davies et al., 1988, 1994; Espanol et al., 1997; Stirling, 1985). Host populations can rapidly evolve resistance (Channer & Gowen, 1992): In Tzortzakakis et al. (1996), *M. javanica* evolved resistance to a population of *P. penetrans *after four generations of selection. Data on adaptation of *P. penetrans* to new host genotypes are limited. Nonetheless, Channer and Gowen (1992) suggest that *P. penetrans* populations can evolve increased attachment to a novel host genotype and reduced attachment to the ancestral host genotype within a single growing season.

Negative frequency‐dependent selection should manifest as rapid and continual temporal change in host specificity of *P. penetrans.* Moreover, trajectories of change should be highly localized in space, such that sites diverge from one another at the scale of host dispersal. Finally, no genotype of *P. penetrans *should be universally more fit; rather, parasites from different plots should differ in the genotype that is most fit at any given point in time. The results of this study are consistent with these patterns: We detected rapid and continual change in host specificity, with trajectories that diverged across sampled plots (Figure 4, Table 2). The results also suggest that no genotype of *P. penetrans *was universally more fit: Averaged across fields, attachment rates were approximately equivalent across clonal host lines (Figure 3, Table 1).

### 4.2 Implications for biological control strategies

Regardless of the driving force, what are the implications of these results for the use of *P. penetrans *as a biological control agent? Clearly, host specificity of *P. penetrans *can change rapidly, as evidenced by substantial variation in specificity in space and time. If the genetic composition of *Meloidogyne *populations varies in space and time, and there is a genetic basis to infection, we expect significant variation in the efficacy of different *P. penetrans* genotypes in controlling a *Meloidogyne *population. Applying a mixture of *P. penetrans *genotypes may overcome this problem: Channer and Gowen (1992) showed that single isolates of *P. penetrans *varied substantially in their ability to attach to different host lineages, while mixtures of four to five parasites isolates showed little variation between host lineages (see also Tzortzakakis & Gowen, 1994). If negative frequency‐dependent selection drives change in specificity in this system, we expect that a population of *P. penetrans *could adapt to infect the most common host genotypes present in a field, given sufficient genetic diversity and a lag time. Indeed, the literature commonly refers to a lag time in the efficacy of *P. penetrans*, with substantial suppression of *Meloidogyne* evident a few years after first application to the field (Chen & Dickson, 1998; Chen, Dickson, McSorley, Mitchell, & Hewlett, 1996; Oostendorp et al., 1991; Timper, 2009). This lag time may reflect adaptation of the *P. penetrans *population to local hosts. An alternate, though not mutually exclusive, explanation is that the lag time reflects the build‐up of endospores to suppressive densities (reviewed in Hufbauer & Roderick, 2005).

Comparison of the crop rotation treatments suggests a conflict between different pest management strategies. Crop rotation reduced attachment rates by nearly 50% relative to continuous planting (Figure 2). The substantial treatment effect, independent of assayed host line, indicates differences in parasite abundance between treatments rather than differences in specificity (Table 1). Though both peanut and soybean are good hosts to *M. arenaria*, nematode reproduction is tenfold greater on peanut than soybean (Noe, 1991). Hence, rotation with soybean can suppress densities of *M. arenaria* (Rodríguez‐Kábana, Robertson, Backman, & Ivey, 1988), in turn suppressing densities of *P. penetrans*. Temporal trajectories of *P. penetrans *specificity were also less divergent across rotation plots than across continuous peanut plots. One possible explanation for this is that, for rotation plots, there was simply less variation in attachment rates to explain because of the low attachment rates in the bioassay. These observations raise an interesting problem for pest management: Interventions, like crop rotation, that reduce the density of *Meloidogyne* hosts may also suppress *P. penetrans* densities, reducing its efficacy in biological control (Madulu, Trudgill, & Phillips, 1994; Timper, 2009; Timper et al., 2001). This finding demonstrates a potential cost of using specific biological control agents that cannot maintain their population sizes on alternative hosts (Fagan et al., 2002).

### 4.3 Conclusion

We have demonstrated rapid change in host specificity in a biological control system. Above, we hypothesize that this change in host specificity reflects adaptation to locally common host genotypes, consistent with host–parasite coevolution. This hypothesis remains to be tested. It predicts that, with sufficient genetic variation, the biological control agent should improve following its initial application, becoming more effective at limiting the population density of its host.

## CONFLICT OF INTEREST

None Declared.

## Data Availability

Data and analysis scripts for this study are available from the Dryad Digital Repository: https://doi.org/10.5061/dryad.0pv6570

## References

[eva12750-bib-0001] Bell, G. (1982). The masterpiece of nature: The evolution and genetics of sexuality. Berkeley, CA: University of California Press.

[eva12750-bib-0002] Bird, A. (1986). The influence of the actionmycete, *Pasteuria penetrans*, on the host–parasite relationship of the plant‐parasitic nematode, Meloidogyne javanica. Parasitology, 93(3), 571–580. 10.1017/S0031182000081270

[eva12750-bib-0003] Bird, A. , & Brisbane, P. (1988). The influence of *Pasteuria penetrans *in field soils on the reproduction of root‐knot nematodes. Revue De Nématologie, 11(1), 75–81.

[eva12750-bib-0004] Blanc‐Mathieu, R. , Perfus‐Barbeoch, L. , Aury, J.‐M. , Da Rocha, M. , Gouzy, J. , Sallet, E. , … Danchin, E. G. J. (2017). Hybridization and polyploidy enable genomic plasticity without sex in the most devastating plant‐parasitic nematodes. PLoS Genetics, 13(6), e1006777 10.1371/journal.pgen.1006777. 28594822 PMC5465968

[eva12750-bib-0005] Boettner, G. H. , Elkinton, J. S. , & Boettner, C. J. (2000). Effects of a biological control introduction on three nontarget native species of saturniid moths. Conservation Biology, 14(6), 1798–1806. 10.1046/j.1523-1739.2000.99193.x 35701905

[eva12750-bib-0006] Brodeur, J. (2012). Host specificity in biological control: Insights from opportunistic pathogens. Evolutionary Applications, 5(5), 470–480. 10.1111/j.1752-4571.2012.00273.x. 22949922 PMC3407865

[eva12750-bib-0007] Brown, S. , Kepner, J. , & Smart, G. (1985). Increased crop yields following application of *Bacillus penetrans *to field plots infested with *Meloidogyne incognita* . Soil Biology and Biochemistry, 17(4), 483–486. 10.1016/0038-0717(85)90014-8

[eva12750-bib-0008] CABI (2017). *Meloidogyne arenaria* (peanut root‐knot nematode) from CAB International. Retrieved from https://www.cabi.org/isc/datasheet/33233

[eva12750-bib-0009] Carius, H. J. , Little, T. J. , & Ebert, D. (2001). Genetic variation in a host‐parasite association: Potential for coevolution and frequency‐dependent selection. Evolution, 55(6), 1136–1145. 10.1111/j.0014-3820.2001.tb00633.x 11475049

[eva12750-bib-0010] Castagnone‐Sereno, P. , Danchin, E. G. , Perfus‐Barbeoch, L. , & Abad, P. (2013). Diversity and evolution of root‐knot nematodes, genus *Meloidogyne*: New insights from the genomic era. Annual Review of Phytopathology, 51, 203–220. 10.1146/annurev-phyto-082712-102300 23682915

[eva12750-bib-0011] Chaboudez, P. , & Burdon, J. (1995). Frequency‐dependent selection in a wild plant‐pathogen system. Oecologia, 102(4), 490–493. 10.1007/BF00341361 28306892

[eva12750-bib-0012] Channer, A. G. D. R. , & Gowen, S. R. (1992). Selection for increased host resistance and increased pathogen specificity in the *Meloidogyne‐Pasteuria penetrans* interaction. Fundamental and Applied Nematology, 15(4), 331–339.

[eva12750-bib-0013] Chen, Z. , & Dickson, D. (1998). Review of *Pasteuria penetrans*: Biology, ecology, and biological control potential. Journal of Nematology, 30(3), 313. 19274225 PMC2620303

[eva12750-bib-0014] Chen, Z. , Dickson, D. W. , McSorley, R. , Mitchell, D. J. , & Hewlett, T. E. (1996). Suppression of *Meloidogyne arenaria* race 1 by soil application of endospores of *Pasteuria penetrans* . Journal of Nematology, 28(2), 159–168. 19277131 PMC2619680

[eva12750-bib-0015] Chitwood, D. J. , & Perry, R. N. (2009). Reproduction, physiology and biochemistry In Perry R., Moens M., & Starr J. (Eds.), Root‐Knot Nematodes (pp. 182–200). Wallingford: CAB International.

[eva12750-bib-0016] R Core Team (2013). R: A language and environment for statistical computing. Vienna, Austria: R Foundation for Statistical Computing http://www.R-project.org

[eva12750-bib-0017] Davies, K. (1994). In vitro recognition of a 190 kda putative attachment receptor from the cuticle of *Meloidogyne javanica* by *Pasteuria penetrans *spore extract. Biocontrol Science and Technology, 4(3), 367–374.

[eva12750-bib-0018] Davies, K. (2009). Understanding the interaction between an obligate hyperparasitic bacterium, *Pasteuria penetrans* and its obligate plant‐parasitic nematode host, *Meloidogyne* spp. Advances in Parasitology *,* 68, 211–245. 19289196 10.1016/S0065-308X(08)00609-X

[eva12750-bib-0019] Davies, K. , Kerry, B. , & Flynn, C. (1988). Observations on the pathogenicity of *Pasteuria penetrans*, a parasite of root‐knot nematodes. Annals of Applied Biology, 112(3), 491–501. 10.1111/j.1744-7348.1988.tb02086.x

[eva12750-bib-0020] Davies, K. , Redden, M. , & Pearson, T. (1994). Endospore heterogeneity in *Pasteuria penetrans* related to adhesion to plant‐parasitic nematodes. Letters in Applied Microbiology, 19, 370–373. 10.1111/j.1472-765X.1994.tb00478.x

[eva12750-bib-0021] Duneau, D. , & Ebert, D. (2012). The role of moulting in parasite defence. Proceedings of the Royal Society B: Biological Sciences, 279(1740), 3049–3054. 10.1098/rspb.2012.0407. PMC3385484 22496187

[eva12750-bib-0022] Duneau, D. , Luijckx, P. , Ben‐Ami, F. , Laforsch, C. , & Ebert, D. (2011). Resolving the infection process reveals striking differences in the contribution of environment, genetics and phylogeny to host‐parasite interactions. BMC Biology, 9(1), 11 10.1186/1741-7007-9-11 21342515 PMC3052238

[eva12750-bib-0023] Duponnois, R. , Fargette, M. , Fould, S. , Thioulouse, J. , & Davies, K. G. (2000). Diversity of the bacterial hyperparasite *Pasteuria penetrans* in relation to root‐knot nematodes (*Meloidogyne* spp.) control on *Acacia holosericea* . Nematology, 2(4), 435–442. 10.1163/156854100509295

[eva12750-bib-0024] Dwyer, G. , Levin, S. A. , & Buttel, L. (1990). A simulation model of the population dynamics and evolution of myxomatosis. Ecological Monographs, 60(4), 423–447. 10.2307/1943014

[eva12750-bib-0025] Ebert, D. , Duneau, D. , Hall, M. D. , Luijckx, P. , Andras, J. P. , Du Pasquier, L. , & Ben‐Ami, F. (2016). A population biology perspective on the stepwise infection process of the bacterial pathogen *Pasteuria ramosa* in *Daphnia* In Rollinson D., & Stothard J. R. (Eds.), Advances in parasitology, Vol. 91 (pp. 265–310). Amsterdam, Netherlands: Academic Press. 10.1016/bs.apar.2015.10.001 27015951

[eva12750-bib-0026] Ebert, D. , Rainey, P. B. , Embley, T. , & Scholz, D. (1996). Development, life cycle, ultrastructure, and phylogenetic position of *Pasteuria ramosa *Metchnikoff 1888: Rediscovery of an obligate endoparasite of *Daphnia magna *Straus. Philosophical Transactions of the Royal Society B: Biological Sciences, 351, 1689–1701.

[eva12750-bib-0027] Engelstadter, J. , & Bonhoeffer, S. (2009). Red Queen dynamics with non‐standard fitness interactions. PLoS Computational Biology, 5(8), e1000469 10.1371/journal.pcbi.1000469 19680432 PMC2715217

[eva12750-bib-0028] Espanol, M. , Verdejo‐Lucas, S. , Davies, K. G. , & Kerry, B. R. (1997). Compatibility Between Pasteuria penetrans Isolates and Meloidogyne Populations from Spain. Biocontrol Science and Technology, 7(2), 219–230. 10.1080/09583159730910.

[eva12750-bib-0029] Fagan, W. F. , Lewis, M. A. , Neubert, M. G. , & van den Driessche, P. (2002). Invasion theory and biological control. Ecology Letters, 5, 148–157. 10.1046/j.1461-0248.2002.0_285.x

[eva12750-bib-0031] Gandon, S. (2002). Local adaptation and the geometry of host–parasite coevolution. Ecology Letters, 5(2), 246–256. 10.1046/j.1461-0248.2002.00305.x

[eva12750-bib-0032] Gandon, S. , & Michalakis, Y. (2002). Local adaptation, evolutionary potential and host–parasite coevolution: Interactions between migration, mutation, population size and generation time. Journal of Evolutionary Biology, 15(3), 451–462. 10.1046/j.1420-9101.2002.00402.x.

[eva12750-bib-0033] Giannakou, I. , Pembroke, B. , Gowen, S. , & Davies, K. (1997). Effects of long term storage and above normal temperatures on spore adhesion of *Pasteuria penetrans *and infection of the root‐knot nematode *Meloidogyne javanica* . Nematologica, 43(2), 185–192. 10.1163/004825997X00051

[eva12750-bib-0034] Greathead, D. (1995). Benefits and risks of classical biological control In Hokkanen H., & Lynch J. (Eds.), Biological control: benefits and risks (pp. 53–63). Cambridge: Cambridge University Press.

[eva12750-bib-0035] Guillebeau, P. (2009). Georgia pest control handbook. Athens, GA: Cooperative Extension Service, University of Georgia, College of Agricultural and Environmental Sciences.

[eva12750-bib-0036] Hajek, A. E. , Humber, R. A. , & Elkinton, J. S. (1995). Mysterious Origin of *Entomphaga maimaiga *in North America. American Entomologist, 41(1), 31–43.

[eva12750-bib-0037] Haldane, J. B. S. (1949). Disease and evolution. La Ricerca Scientifica, 19(Suppl. 1), 68–76.

[eva12750-bib-0038] Hamilton, W. (1980). Sex versus non‐sex versus parasite. Oikos, 35(2), 282–290. 10.2307/3544435

[eva12750-bib-0039] Hamilton, W. , Axelrod, R. , & Tanese, R. (1990). Sexual reproduction as an adaptation to resist parasites (a review). Proceedings of the National Academy of Sciences, 87(9), 3566–3573. 10.1073/pnas.87.9.3566 PMC53943 2185476

[eva12750-bib-0040] Hopper, K. , Roush, R. , & Powell, W. (1993). Management of genetics of biological‐control introductions. Annual Review of Entomology, 38(1), 27–51. 10.1146/annurev.en.38.010193.000331

[eva12750-bib-0041] Hufbauer, R. A. , & Roderick, G. K. (2005). Microevolution in biological control: Mechanisms, patterns, and processes. Biological Control, 35(3), 227–239. 10.1016/j.biocontrol.2005.04.004.

[eva12750-bib-0042] Hutson, V. , & Law, R. (1981). Evolution of recombination in populations experiencing frequency‐dependent selection with time delay. Proceedings of the Royal Society B: Biological Sciences, 213, 345–359. 10.1098/rspb.1981.0070

[eva12750-bib-0043] Ingram, E. , & Rodriguez‐Kabana, R. (1980). Nematodes parasitic on peanuts in Alabama and evaluation of methods for detection and study of population dynamics. Nematropica, 10(1), 21–30.

[eva12750-bib-0044] Jaenike, J. (1978). An hypothesis to account for the maintenance of sex within populations. Evolutionary Theory, 3(4), 191–194.

[eva12750-bib-0045] Jenkins, W. R. (1964). A rapid centrifugal‐flotation technique for separating nematodes from soil. Plant Disease Reporter, 48, 692–692.

[eva12750-bib-0046] Jones, J. T. , Haegeman, A. , Danchin, E. G. , Gaur, H. S. , Helder, J. , Jones, M. G. , … Wesemael, W. M. (2013). Top 10 plant‐parasitic nematodes in molecular plant pathology. Molecular Plant Pathology, 14(9), 946–961. 10.1111/mpp.12057 23809086 PMC6638764

[eva12750-bib-0047] Joseph, S. , Schmidt, L. M. , Danquah, W. B. , Timper, P. , & Mekete, T. (2017). Genotyping of single spore isolates of a *Pasteuria penetrans *population occurring in Florida using SNP‐based markers. Journal of Applied Microbiology, 122(2), 389–401. 10.1111/jam.13345. 27862724

[eva12750-bib-0048] Koskella, B. , & Lively, C. M. (2009). Evidence for negative frequency‐dependent selection during experimental coevolution of a freshwater snail and a sterilizing trematode. Evolution, 63(9), 2213–2221. 10.1111/j.1558-5646.2009.00711.x 19473396

[eva12750-bib-0049] Kruitwagen, A. , Beukeboom, L. W. , & Wertheim, B. (2018). Optimization of native biocontrol agents, with parasitoids of the invasive pest *Drosophila suzukii *as an example. Evolutionary Applications, 11(9), 1473–1497. 10.1111/eva.12648. 30344621 PMC6183459

[eva12750-bib-0050] Le Masurier, A. , & Waage, J. (1993). A comparison of attack rates in a native and an introduced population of the parasitoid *Cotesia glomerata* . Biocontrol Science and Technology, 3(4), 467–474.

[eva12750-bib-0051] Liu, C. , Timper, P. , Ji, P. , Mekete, T. , & Joseph, S. (2017). Influence of root exudates and soil on attachment of *Pasteuria penetrans* to *Meloidogyne arenaria* . Journal of Nematology, 49(3), 304–310. 10.21307/jofnem-2017-076 29062153 PMC5644923

[eva12750-bib-0052] Lively, C. M. , & Dybdahl, M. F. (2000). Parasite adaptation to locally common host genotypes. Nature, 405(6787), 679–681. 10864323 10.1038/35015069

[eva12750-bib-0053] Lopes, E. A. , Orr, J. N. , & Blok, V. C. (2018). Does soil warming affect the interaction between *Pasteuria penetrans* and *Meloidogyne javanica* in tomato plants? Plant Pathology, 67(8), 1777–1783. 10.1111/ppa.12877.

[eva12750-bib-0054] Louda, S. M. , Kendall, D. , Connor, J. , & Simberloff, D. (1997). Ecological effects of an insect introduced for the biological control of weeds. Science, 277(5329), 1088–1090.

[eva12750-bib-0055] Louda, S. M. , Pemberton, R. , Johnson, M. , & Follett, P. (2003). Nontarget effects—the Achilles' heel of biological control? Retrospective analyses to reduce risk associated with biocontrol introductions. Annual Review of Entomology, 48(1), 365–396. 10.1146/annurev.ento.48.060402.102800 12208812

[eva12750-bib-0056] Luijckx, P. , Ben‐Ami, F. , Mouton, L. , Du Pasquier, L. , & Ebert, D. (2011). Cloning of the unculturable parasite *Pasteuria ramosa* and its *Daphnia* host reveals extreme genotype–genotype interactions. Ecology Letters, 14(2), 125–131. 10.1111/j.1461-0248.2010.01561.x. 21091597

[eva12750-bib-0057] Luijckx, P. , Fienberg, H. , Duneau, D. , & Ebert, D. (2012). Resistance to a bacterial parasite in the crustacean *Daphnia magna* shows Mendelian segregation with dominance. Heredity, 108(5), 547–551. 10.1038/hdy.2011.122. 22167056 PMC3330695

[eva12750-bib-0058] Luijckx, P. , Fienberg, H. , Duneau, D. , & Ebert, D. (2013). A matching‐allele model explains host resistance to parasites. Current Biology, 23(12), 1085–1088. 10.1016/j.cub.2013.04.064 23707426

[eva12750-bib-0059] Madulu, J. , Trudgill, D. , & Phillips, M. (1994). Rotational management of *Meloidogyne javanica *and effects on *Pasteuria penetrans* and tomato and tobacco yields. Nematologica, 40(1), 438–455. 10.1163/003525994X00319

[eva12750-bib-0060] Mangiafico, S. (2015). An R Companion for the Handbook of Biological Statistics, version 1.3.2. http://rcompanion.org

[eva12750-bib-0061] Mani, A. (1988). Studies on the bacterial parasite *Pasteuria penetrans*: I. Spore viability after storage. II. Culture on citrus nematode *Tylenchulus semipenetrans* . International Nematology Network Newsletter, 5, 24–25.

[eva12750-bib-0062] Mankau, R. (1975). *Bacillus penetrans *n. comb. causing a virulent disease of plant‐parasitic nematodes. Journal of Invertebrate Pathology, 26, 333–339. 10.1016/0022-2011(75)90231-1

[eva12750-bib-0063] Mankau, R. (1980). Biological control of nematode pests by natural enemies. Annual Review of Phytopathology, 18, 415–440. 10.1146/annurev.py.18.090180.002215

[eva12750-bib-0064] Mankau, R. , & Imbriani, J. (1975). The life cycle of an endoparasite in some tylenchid nematodes. Nematologica, 21(1), 89–94. 10.1163/187529275X00383

[eva12750-bib-0065] Mankau, R. , & Prasad, N. (1972). Possibilities and problems in the use of a sporozoan endoparasite for biological control of plant parasitic nematodes. Nematropica, 2, 7–8.

[eva12750-bib-0066] McEvoy, P. (1996). Host specificity and biological pest control. BioScience, 46(6), 401–405. 10.2307/1312873

[eva12750-bib-0067] McFadden, D. (1974). Conditional logit analysis of qualitative choice behavior In Zarembka P. (Ed.), Frontiers in Econometrics (pp. 105–142). New York, NY: Academic Press.

[eva12750-bib-0068] McFadden, D. (1979). Quantitative methods for analyzing travel behavior of individuals: Some recent developments In Hensher D., & Stopher P. (Eds.), Behavioral travel modeling (pp. 279–318). London: Croom Helm.

[eva12750-bib-0069] Metchnikoff, E. (1888). Pasteuria ramosa, un représentant des bactéries à division longitudinale. Annales De L'institut Pasteur, 2, 165–170.

[eva12750-bib-0070] Metzger, C. M. , Luijckx, P. , Bento, G. , Mariadassou, M. , & Ebert, D. (2016). The Red Queen lives: Epistasis between linked resistance loci. Evolution, 70(2), 480–487. 10.1111/evo.12854. 26763092

[eva12750-bib-0071] Meyer, S. E. , Nelson, D. L. , Clement, S. , Waters, J. , Stevens, M. , & Fairbanks, D. (2005). Genetic variation in *Ustilago bullata*: Molecular genetic markers and virulence on *Bromus tectorum* host lines. International Journal of Plant Sciences, 166(1), 105–115.

[eva12750-bib-0072] Moens, M. , Perry, R. , & Starr, J. (2009). *Meloidogyne *species ‐ a diverse group of novel and important plant parasites In Perry R., Moens M., & Starr J. (Eds.), Root‐knot nematodes. Wallingford: CAB International.

[eva12750-bib-0073] Motsinger, R. , Crawford, J. , & Thompson, S. (1976). Nematode survey of peanuts and cotton in southwest Georgia. Peanut Science, 3(2), 72–74. 10.3146/i0095-3679-3-2-5

[eva12750-bib-0074] Nicol, J. M. , Turner, S. J. , Coyne, D. L. , Nijs, L. D. , Hockland, S. , & Maafi, Z. T. (2011). Current nematode threats to world agriculture In Jones J., Gheysen G., & Fenoll C. (Eds.), Genomics and molecular genetics of plant‐nematode interactions (pp. 21–43). Dordrecht: Springer Science and Business Media.

[eva12750-bib-0075] Noe, J. (1991). Development of *Meloidogyne arenaria o*n peanut and soybean under two temperature cycles. Journal of Nematology, 23(4), 468. 19283157 PMC2619182

[eva12750-bib-0076] Onkendi, E. , Kariukib, G. , Maraisc, M. , & Molelekia, L. N. (2014). The threat of root‐knot nematodes (*Meloidogyne* spp.) in Africa: A review. Plant Pathology, 63, 727–737.

[eva12750-bib-0077] Oostendorp, M. , Dickson, D. , & Mitchell, D. (1990). Host range and ecology of isolates of *Pasteuria* spp. from the southeastern United States. Journal of Nematology, 22(4), 525–531. 19287753 PMC2619090

[eva12750-bib-0078] Oostendorp, M. , Dickson, D. , & Mitchell, D. (1991). Population development of *Pasteuria penetrans *on *Meloidogyne arenaria* . Journal of Nematology, 23(1), 58–64. 19283094 PMC2619133

[eva12750-bib-0079] Parker, M. A. (1985). Local population differentiation for compatibility in an annual legume and its host‐specific fungal pathogen. Evolution, 39(4), 713–723. 10.2307/2408672. 28561354

[eva12750-bib-0080] Parker, M. A. (1994). Pathogens and sex in plants. Evolutionary Ecology, 8(5), 560–584. 10.1007/bf01238258.

[eva12750-bib-0081] Phani, V. , & Rao, U. (2018). Revisiting the life‐cycle of *Pasteuria penetrans* infecting *Meloidogyne incognita* under soil‐less medium, and effect of streptomycin sulfate on its development. Journal of Nematology, 50(2), 91–98. 30451430 10.21307/jofnem-2018-022 PMC6909293

[eva12750-bib-0082] Preston, J. F. , Dickson, D. W. , Maruniak, J. E. , Nong, G. , Brito, J. A. , Schmidt, L. M. , & Giblin‐Davis, R. M. (2003). *Pasteuria *spp.: Systematics and phylogeny of these bacterial parasites of phytopathogenic nematodes. Journal of Nematology, 35(2), 198–207. 19265995 PMC2620627

[eva12750-bib-0083] Prot, J.‐C. , & Netscher, C. (1979). Influence of movement of juveniles on detection of fields infested with *Meloidogyne* In Lamberti F., & Taylor C. E. (Eds.), Root‐knot nematodes (Meloidogyne species): Systematics, biology and control. New York, NY: Academic Press.

[eva12750-bib-0084] Roderick, G. K. , Hufbauer, R. , & Navajas, M. (2012). Evolution and biological control. Evolutionary Applications, 5, 419–423. 10.1111/j.1752-4571.2012.00281.x 22949918 PMC3407861

[eva12750-bib-0085] Roderick, G. K. , & Navajas, M. (2003). Genes in new environments: Genetics and evolution in biological control. Nature Reviews Genetics, 4(11), 889 10.1038/nrg1201 14634636

[eva12750-bib-0086] Rodríguez‐Kábana, R. , Robertson, D. G. , Backman, P. A. , & Ivey, H. (1988). Soybean‐peanut rotations for the management of *Meloidogyne arenaria* . Journal of Nematology, 20(Annals 2), 81–85. 19290309 PMC2618874

[eva12750-bib-0087] Salt, G. , & van den Bosch, R. (1967). The defense reactions of three species of *Hypera *(Coleoptera, Curculionidae) to an ichneumon wasp. Journal of Invertebrate Pathology, 9(2), 164–177. 10.1016/0022-2011(67)90005-5

[eva12750-bib-0088] Sasser, J. , & Carter, C. (1985). Overview of the international meloidogyne project 1975–1984 In Sasser J., & Carter C. (Eds.), An advanced treatise on meloidogyne: biology and control (Vol. 1, pp. 19–24). Raleigh, NC: North Carolina State University Graphics.

[eva12750-bib-0089] Sasser, J. (1977). Worldwide dissemination and importance of the root‐knot nematodes, *Meloidogyne *spp. Journal of Nematology, 9(1), 26–29. 19305566 PMC2620215

[eva12750-bib-0090] Sasser, J. , Eisenback, J. D. , & Carter, C. (1983). The International *Meloidogyne *Project ‐ its goals and accomplishments. Annual Review of Phytopathology, 21, 271–288. 10.1146/annurev.py.21.090183.001415

[eva12750-bib-0091] Sayre, R. , & Starr, M. (1985). *Pasteuria penetrans *(ex Thome, 1940) nom. rev., comb, n., sp. n., a mycelial and endospore‐forming bacterium parasitic in plant‐parasitic nematodes. Proceedings of the Helminthological Society of Washington, 52(2), 149–165.

[eva12750-bib-0092] Sayre, R. , & Wergin, W. (1977). Bacterial parasite of a plant nematode: Morphology and ultrastructure. Journal of Bacteriology, 129(2), 1091–1101. 838678 10.1128/jb.129.2.1091-1101.1977 PMC235050

[eva12750-bib-0093] Secord, D. , & Kareiva, P. (1996). Perils and pitfalls in the host specificity paradigm. BioScience, 46(6), 448–453. 10.2307/1312879

[eva12750-bib-0094] Sicard, D. , Michalakis, Y. , Dron, M. , & Neema, C. (1997). Genetic diversity and pathogenic variation of *Colletotrichum lindemuthianum* in the three centers of diversity of its host, *Phaseolus Vulgaris* . Phytopathology, 87(8), 807–813. 18945048 10.1094/PHYTO.1997.87.8.807

[eva12750-bib-0095] Simberloff, D. , & Stiling, P. (1996). How risky is biological control? Ecology, 77(7), 1965–1974.

[eva12750-bib-0096] Spaull, V. (1984). Observations on *Bacillus penetrans *infecting *Meloidogyne* in sugarcane fields in South Africa. Revue De Nématologie, 7(3), 277–282.

[eva12750-bib-0097] Starr, J. , & Morgan, E. (2002). Management of the peanut root‐knot nematode, *Meloidogyne arenaria*, with host resistance. Plant Health Progress, 3, 13 10.1094/PHP-2002-1121-01-HM.

[eva12750-bib-0098] Starr, M. , & Sayre, R. (1988). *Pasteuria thornei sp. nov.* and *Pasteuria penetrans sensu stricto emend*., mycelial and endospore‐forming bacteria parasitic, respectively, on plant‐parasitic nematodes of the genera *Pratylenchus* and *Meloidogyne* . Annales De L'institut Pasteur/Microbiologie, 139(1), 11–31. 10.1016/0769-2609(88)90094-4 3382544

[eva12750-bib-0099] Stirling, G. (1984). Biological control of *Meloidogyne javanica *with *Bacillus penetrans* . Phytopathology, 74(1), 55–60.

[eva12750-bib-0100] Stirling, G. (1985). Host specificity of *Pasteuria penetrans *within the genus *Meloidogyne* . Nematologica, 31, 203–209. 10.1163/187529285X00265

[eva12750-bib-0101] Tarr, D. E. K. (2012). Distribution and characteristics of ABFs, cecropins, nemapores, and lysozymes in nematodes. Developmental & Comparative Immunology, 36(3), 502–520. 10.1016/j.dci.2011.09.007. 21978453

[eva12750-bib-0102] Thrall, P. H. , Laine, A.‐L. , Ravensdale, M. , Nemri, A. , Dodds, P. N. , Barrett, L. G. , & Burdon, J. J. (2012). Rapid genetic change underpins antagonistic coevolution in a natural host‐pathogen metapopulation. Ecology Letters, 15(5), 425–435. 10.1111/j.1461-0248.2012.01749.x. 22372578 PMC3319837

[eva12750-bib-0103] Timper, P. (2009). Population dynamics of *Meloidogyne arenaria* and *Pasteuria penetrans *in a long‐term crop rotation study. Journal of Nematology, 41(4), 291–299. 22736828 PMC3381465

[eva12750-bib-0104] Timper, P. , Minton, N. , Johnson, A. , Brenneman, T. , Culbreath, A. , Burton, G. , … Gascho, G. (2001). Influence of cropping systems on stem rot (*Sclerotium rolfsii*), *Meloidogyne arenaria,* and the nematode antagonist *Pasteuria penetrans* in peanut. Plant Disease, 85(7), 767–772. 30823204 10.1094/PDIS.2001.85.7.767

[eva12750-bib-0105] Triantaphyllou, A. (1985). Cytogenetics, cytotaxonomy and phylogeny of root‐knot nematodes In Sasser J., & Carter C. (Eds.), An advanced treatise on meliodogyne, Vol. 1 (pp. 113–126). Raleigh, NC: North Carolina State University Graphics.

[eva12750-bib-0106] Triantaphyllou, A. (1991). Further studies on the role of polyploidy in the evolution of *Meloidogyne* . Journal of Nematology, 23(2), 249. 19283121 PMC2619150

[eva12750-bib-0107] Trudgill, D. , & Blok, V. (2001). Apomictic, polyphagous root‐knot nematodes: Exceptionally successful and damaging biotrophic root pathogens. Annual Review of Phytopathology, 39, 53–77. 10.1146/annurev.phyto.39.1.53 11701859

[eva12750-bib-0108] Trudgill, D. , Bala, G. , Blok, V. , Daudi, A. , Davies, K. G. , Gowen, S. R. , … Voyoukallou, E. (2000). The importance of tropical root‐knot nematodes (*Meloidogyne *spp.) and factors affecting the utility of *Pasteuria penetrans *as a biological control agent. Nematology, 2(8), 823–845.

[eva12750-bib-0109] Tzortzakakis, E. , & Gowen, S. (1994). Resistance of a population of *Meloidogyne *spp. to parasitism by the obligate parasite *Pasteuria penetrans* . Nematologica, 40(1), 258–266.

[eva12750-bib-0110] Tzortzakakis, E. , Gowen, S. R. , & Goumas, D. (1996). Decreased ability of *Pasteuria penetrans* spores to attack to successive generations of *Meloidogyne javanica* . Fundamental and Applied Nematology, 19(2), 201–204.

[eva12750-bib-0111] van Klinken, R. , & Edwards, O. (2002). Is host‐specificity of weed biological control agents likely to evolve rapidly following establishment? Ecology Letters, 5, 590–596.

[eva12750-bib-0112] Waage, J. K. (2001). Indirect ecological effects in biological control: The challenge and the opportunity In Wajnberg E., Scott J., & Quimby P. (Eds.), Evaluating indirect ecological effects of biological control (pp. 744–12). Wallingford: CAB International.

[eva12750-bib-0113] Wang, Y.‐G. , & Carey, V. (2003). Working correlation structure misspecification, estimation and covariate design: Implications for generalised estimating equations performance. Biometrika, 90(1), 29–41. 10.1093/biomet/90.1.29

[eva12750-bib-0114] Wheeler, T. , & Starr, J. (1987). Incidence and economic importance of plant‐parasitic nematodes on peanut in Texas. Peanut Science, 14(2), 94–96. 10.3146/i0095-3679-14-2-11

[eva12750-bib-0115] Williams, A. B. , Stirling, G. , Hayward, A. , & Perry, J. (1989). Properties and attempted culture of *Pasteuria penetrans*, a bacterial parasite of root‐knot nematode (*Meloidogyne javanica*). Journal of Applied Microbiology, 67(2), 145–156.

[eva12750-bib-0116] Wolinska, J. , & Spaak, P. (2009). The cost of being common: Evidence from natural *Daphnia* populations. Evolution, 63(7), 1893–1901. 19228186 10.1111/j.1558-5646.2009.00663.x

[eva12750-bib-0117] Ziegler, A. , & Vens, M. (2010). Generalized estimating equations: Notes on the choice of the working correlation matrix. Methods of Information in Medicine, 49, 421–425. 10.3414/ME10-01-0026 20871939

[eva12750-bib-0118] Zuur, A. , Ieno, E. , Walker, N. , Saveliev, A. , & Smith, G. (2009). Generalised estimating equations In Zuur A., Ieno E., Walker N., Saveliev A., & Smith G. (Eds.), Mixed effects models and extensions in ecology with R. New York, NY: Springer-Verlag.

