# Supplementary material for: Rapid change in host specificity in a field population of the biological control organism Pasteuria penetrans
Source: Evol Appl. 2018 Dec 31;12(4):744–56. doi: 10.1111/eva.12750 (PMC6439493; doi:10.1111/eva.12750)
Supplement: Supplementary file 1 [file EVA-12-744-s001.docx]

**Supporting Material**

| **Table S1: Variation in endospore attachment rate for each plot** | |
| --- | --- |
| Peanut Plots | |
| \| **Plot A** \| *Df* \| *D* \| *p-value* \| \| --- \| --- \| --- \| --- \| \| Year \| 3 \| 37.40 \| <.001 \| \| Line \| 3 \| 21.13 \| <.001 \| \| Year*Line \| 9 \| 51.13 \| <.001 \| \| Null Deviance \| 399 \| 541.49 \|  \| \| Residual Deviance \| 384 \| 431.83 \|  \| \| *R_2_^L^* = 0.1203 \|  \|  \|  \| | \| **Plot B** \| *Df* \| *D* \| *p-value* \| \| --- \| --- \| --- \| --- \| \| Year \| 3 \| 83.51 \| <.001 \| \| Line \| 3 \| 9.29 \| .026 \| \| Year*Line \| 9 \| 34.38 \| <.001 \| \| Null Deviance \| 399 \| 545.48 \|  \| \| Residual Deviance \| 384 \| 418.31 \|  \| \| *R_2_^L^* = 0.233 \|  \|  \|  \| |
| \| **Plot C** \| *Df* \| *D* \| *p-value* \| \| --- \| --- \| --- \| --- \| \| Year \| 3 \| 21.97 \| <.001 \| \| Line \| 3 \| 4.08 \| .253 \| \| Year*Line \| 9 \| 60.01 \| <.001 \| \| Null Deviance \| 399 \| 543.58 \|  \| \| Residual Deviance \| 384 \| 457.52 \|  \| \| *R_2_^L^* = 0.158 \|  \|  \|  \| | \| **Plot D** \| *Df* \| *D* \| *p-value* \| \| --- \| --- \| --- \| --- \| \| Year \| 3 \| 10.77 \| .013 \| \| Line \| 3 \| 19.71 \| <.001 \| \| Year*Line \| 9 \| 42.46 \| <.001 \| \| Null Deviance \| 399 \| 540.75 \|  \| \| Residual Deviance \| 384 \| 467.80 \|  \| \| *R_2_^L^* = 0.135 \|  \|  \|  \| |
| Rotation Plots | |
| \| **Plot E** \| *Df* \| *D* \| *p-value* \| \| --- \| --- \| --- \| --- \| \| Year \| 3 \| 12.67 \| .005 \| \| Line \| 3 \| 25.15 \| <.001 \| \| Year*Line \| 9 \| 72.91 \| <.001 \| \| Null Deviance \| 399 \| 438.54 \|  \| \| Residual Deviance \| 384 \| 327.81 \|  \| \| *R_2_^L^* = 0.253 \|  \|  \|  \| | \| **Plot F** \| *Df* \| *D* \| *p-value* \| \| --- \| --- \| --- \| --- \| \| Year \| 3 \| 39.26 \| <.001 \| \| Line \| 3 \| 0.68 \| .877 \| \| Year*Line \| 9 \| 24.95 \| .003 \| \| Null Deviance \| 399 \| 492.03 \|  \| \| Residual Deviance \| 384 \| 427.14 \|  \| \| *R_2_^L^* = 0.132 \|  \|  \|  \| |
| \| **Plot G** \| *Df* \| *D* \| *p-value* \| \| --- \| --- \| --- \| --- \| \| Year \| 3 \| 8.33 \| .040 \| \| Line \| 3 \| 17.67 \| <.001 \| \| Year*Line \| 9 \| 38.46 \| <.001 \| \| Null Deviance \| 399 \| 416.41 \|  \| \| Residual Deviance \| 384 \| 351.95 \|  \| \| *R_2_^L^* = 0.350 \|  \|  \|  \| | \| **Plot H** \| *Df* \| *D* \| *p-value* \| \| --- \| --- \| --- \| --- \| \| Year \| 3 \| 41.06 \| <.001 \| \| Line \| 3 \| 25.03 \| <.001 \| \| Year*Line \| 9 \| 50.84 \| <.001 \| \| Null Deviance \| 399 \| 493.67 \|  \| \| Residual Deviance \| 384 \| 376.74 \|  \| \| *R_2_^L^* = 0.237 \|  \|  \|  \| |
| The table presents the results of generalized linear models with the attachment status of an individual nematode (endospores attached or not) as a binomial response variable. A separate model was fit for each plot. | |

| 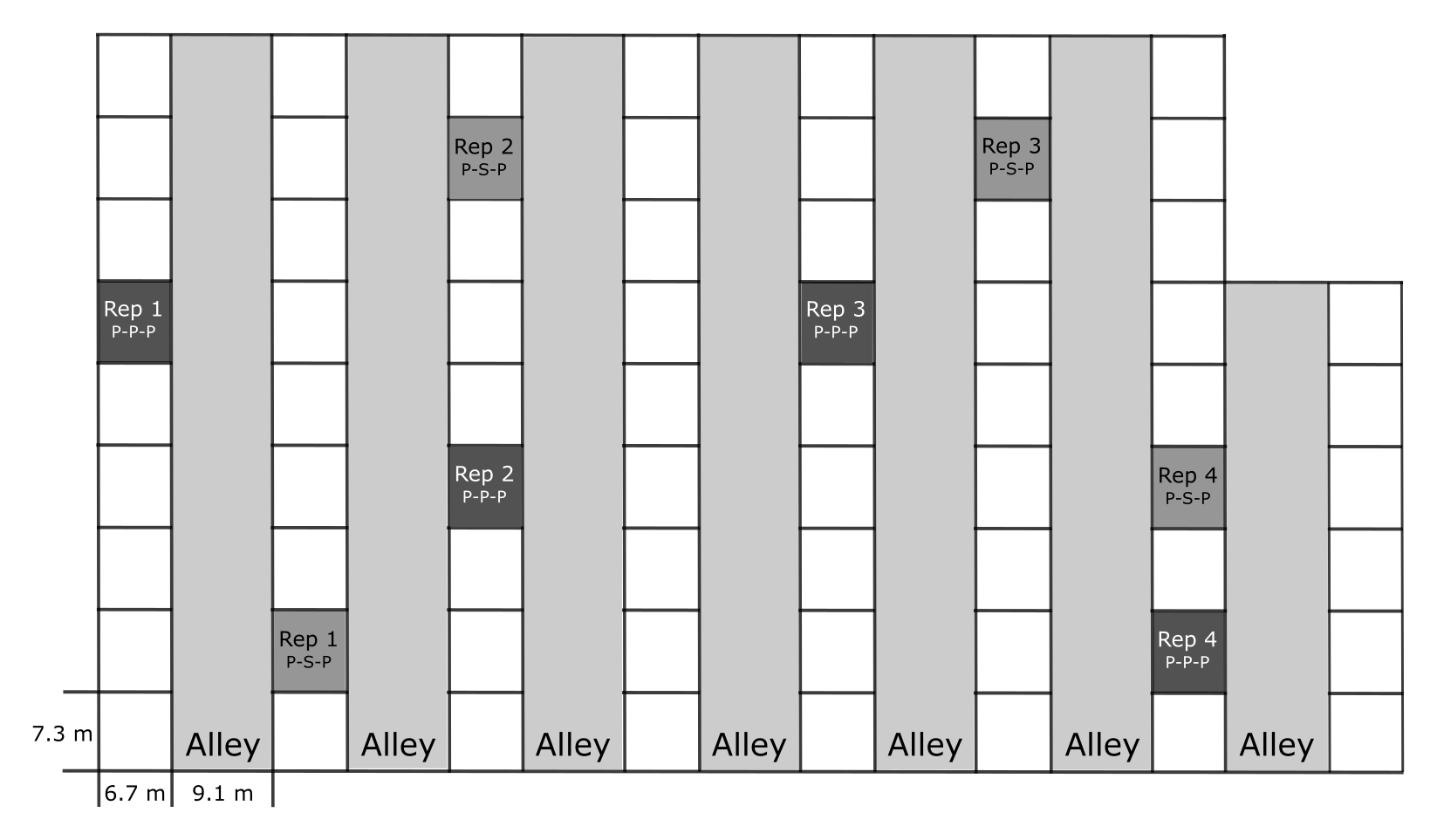 |
| --- |
| **Figure S1: Layout of the study site.**  From 2013 to 2016, eight plots in a 0.77 hectare field were sampled for *Pasteuria penetrans*. The fields were either planted continuously with peanut (P-P-P; 4 replicates) or a rotation of peanut and soybean (P-S-P; 4 replicates). |
